# Letter to the Editor: “Impact of autopsy on clarification of the cause of death in pediatric COVID-19 fatalities”

**DOI:** 10.1186/s41935-022-00301-6

**Published:** 2022-10-20

**Authors:** Josef Finsterer

**Affiliations:** Neurology & Neurophysiology Center, Postfach 20, 1180 Vienna, Austria

**Keywords:** SARS-CoV-2, Vaccination, Rhabdomyolysis, Muscle, Side effect

## Main text

We read with interest the article by Octavius et al. about a systematic review on autopsy findings of 15 pediatric patients from eight studies with a fatal COVID-19 infection (Octavius et al. 2022). Pathologic findings from the heart included diffuse inflammatory infiltrates, myocarditis, cardiomyocyte necrosis, pericarditis, and interstitial edema (Octavius et al. 2022). Histopathological abnormalities of the lungs included alveolar damage, cytopathic changes, thrombi within arterioles and septal capillaries, lung congestion, focal edema, cytopathic changes, and pneumocyte hyperplasia (Octavius et al. 2022). The study is promising but raises concerns that should be discussed.

A limitation of the study is that the cause of death remained unexplained in seven of the fifteen included patients (Octavius et al. 2022). Which is the reason why these patients were autopsied? Particularly, from these seven patients, we should know the clinical course during hospitalization and the medication these patients received for the infection. Is it conceivable that at least some of these patients died from side effects to the applied treatment management?

Apparently, a brain biopsy was performed on six of the 15 included patients (Octavius et al. 2022). What was the indication for a brain biopsy in these patients? One of these patients had meningoencephalitis as the cause of death (Octavius et al. 2022). Was the meningoencephalitis diagnosed intra vitam or only post mortem in this particular patient? If meningitis was already known intra vitam, we should know why the patient was autopsied. Why were brain autopsies not performed on the other nine patients? Knowing the results of cerebral autopsies is crucial as SARS-CoV-2 infections are often complicated by involvement of the brain or the spinal cord (Finsterer et al. 2020) and heart and lung disease is often not the cause of death but the cerebral involvement (Cavalcanti et al. 2020).

Since many patients dying from COVID-19 are in an intensive care unit at the time of death, it is conceivable that in cases where the brain or the spinal cord was not autopsied, neurological deficits were not detected. In sedated and ventilated patients, it is not always easy to recognize whether a neurological deficit had developed as a complication of the infection. In particular, it is difficult to detect conditions such as meningitis, encephalitis, stroke, bleeding, vasculitis, and Guillain–Barre syndrome (GBS) when there is no active search for them or when patients are awake to follow commands or answer questions. A brain biopsy would be mandatory, especially in the seven cases with unclear cause of death.

Surprisingly, the cerebrospinal fluid (CSF) was examined post-mortem in only one patient (Octavius et al. 2022). What was the indication for this? Was that the patient with meningoencephalitis? Why was the CSF not examined post-mortem in all other patients?

Apparently, only eight of the included patients underwent cardiac autopsy. We should be informed of the reasons why not all 15 patient underwent heart autopsy. In myocarditis, we should know the difference between “diffuse inflammatory infiltrate” (2 patients) and “myocarditis” (3 patients) (Octavius et al. 2022). Was there no infiltration in the three patients with myocarditis? Furthermore, an increase in heart weight has been attributed to viral myocarditis (Octavius et al. 2022). We should know if the virus has been definitively detected in cardiomyocytes or the interstitium. Has the myocardium been examined by electron microscopy? The virus is not visible under light microscopy (Leigh et al. 2021).

A further limitation is that it was not specified what is meant with “case reports needed to fulfill the majority of the JBJ” (Octavius et al. 2022). This criterion is unprecise and may lead to bias. We should be informed which and how many of the JBJ had to be fulfilled to be included or excluded from the review. We should know how many JBJ criteria were met by the eight included studies.

Concerning the patient with adrenal carcinoma, we should know if malignancy was detected already intra vitam and if it was the cause of death and not the COVID-19 infection.

Overall, the interesting study has limitations that call the results and their interpretation into question. Clarifying these weaknesses would strengthen the conclusions and could improve the study. As autopsy revealed the cause of death in only half of the patients, it is questionable if autopsy is truly helpful to clarify the cause of death in fatal pediatric COVID-19 cases.

## Data Availability

Not applicable.

## Abbreviations

COVID-19: Coronavirus disease
CSF: Cerebrospinal fluid
GBS: Guillain–Barre syndrome
JBJ: Joanna Briggs Institute’s appraisal checklist
SARS-CoV-2: Severe, acquired respiratory syndrome coronavirus-2
